# Digital Literacy and Patient Satisfaction in Telemedicine Follow-Up With In-Person App Instruction Versus Outpatient Department Follow-Up After Upper-Extremity Surgery: A Randomized Controlled Trial

**DOI:** 10.2196/86918

**Published:** 2026-06-25

**Authors:** Werapat Ngowroongrueng, Sitthiphong Suwannaphisit, Chanon Malaikritsanachalee, Sutee Thaveepunsan, Sirisak Chaitantipongse, Theephop Teeragananan, Kanokporn Imsakul, Supaira Puntumabumrung, Krit Prasittichok

**Affiliations:** 1 Department of Orthopaedics, Faculty of Medicine, Vajira Hospital, Navamindradhiraj University, 681 Samsen Road, Dusit, Bangkok, 10300, Thailand, 66 81 901 0614; 2 Faculty of Medicine Vajira Hospital, Navamindradhiraj University, Bangkok, Thailand

**Keywords:** telemedicine, digital literacy, hand surgery, postoperative care, patient satisfaction, urban areas, Thailand

## Abstract

**Background:**

Telemedicine is increasingly used for postoperative follow-up in orthopedic surgery, but concerns remain regarding whether patients’ digital literacy influences satisfaction with virtual care.

**Objective:**

This study aimed to compare patient satisfaction between telemedicine and in-person outpatient department (OPD) follow-up after upper-extremity surgery and to examine the association between digital literacy and patient satisfaction.

**Methods:**

This single-center, open-label randomized controlled trial enrolled adults undergoing nontraumatic hand or upper-extremity surgery. After trial registration, 70 participants were randomized in a 1:1 ratio to telemedicine (n=35, 50%) or in-person OPD follow-up (n=35, 50%) using block randomization with a fixed block size of 4. All participants received standardized postoperative education at the 2-week visit. Participants in the telemedicine group additionally received brief in-person instruction on the hospital-based Vajira@Home telemedicine app before completing a scheduled video-based follow-up at 6 weeks. Patient satisfaction was assessed at 2 and 6 weeks using a validated 6-item Likert scale questionnaire covering communication, convenience, understanding of care, and overall satisfaction. Digital literacy was measured at baseline using a standardized questionnaire. Between-group comparisons were performed using appropriate parametric or nonparametric tests.

**Results:**

Participants in the telemedicine group were younger than those in the outpatient department (OPD) group (mean 53.3, SD 11.5 years vs mean 59.7, SD 14.2 years) and had higher digital literacy scores (mean 12.0, SD 3.5 vs mean 10.2, SD 2.5). Overall satisfaction scores were high in both groups at 2 and 6 weeks postoperatively (median 5, IQR 5-5 at both time points). No statistically significant differences were observed between groups across any satisfaction domains, including communication (*P*=.27), convenience (*P*=.41), and overall satisfaction (*P*=.35). Satisfaction scores remained stable over time within each group. Digital literacy was not associated with patient satisfaction.

**Conclusions:**

Telemedicine follow-up supported by brief in-person app instruction resulted in patient satisfaction that was not significantly different from traditional in-person outpatient follow-up after upper-extremity surgery. Although age and digital literacy differed modestly between groups, these factors did not influence satisfaction. These findings support telemedicine as an acceptable option for postoperative follow-up in appropriately selected patients.

## Introduction

The rapid growth of telemedicine has transformed health care delivery by enabling remote consultations, expanding accessibility, and reducing travel-related burdens for patients [1-3]. In orthopedic surgery, virtual follow-up represents a practical and increasingly favored alternative to conventional outpatient visits, particularly for postoperative assessment and rehabilitation [4-7]. Previous studies [8,9] have shown that telemedicine can maintain patient safety and satisfaction while reducing health care costs and alleviating clinic overcrowding. However, the effectiveness and acceptability of telemedicine depend not only on technological infrastructure but also on patients’ ability to engage confidently and effectively with digital platforms [10,11].

Digital literacy, defined as the ability to use digital technologies and critically interpret online information, has emerged as a key factor influencing the success of telemedicine [12]. Although smartphone ownership and internet connectivity have expanded globally, disparities in digital competence persist, especially among older adults and individuals with lower educational attainment [13]. In orthopedic practice, such disparities may hinder equitable access to postoperative care and slow the broader adoption of digital health solutions. Prior research [11] has shown that patients with strong health literacy but limited digital literacy often face challenges when navigating telemedicine systems. Nevertheless, the relationship between digital literacy and patient satisfaction in surgical populations, particularly within middle-income countries, remains insufficiently explored.

Thailand, an emerging digital economy in Southeast Asia, provides an appropriate context in which to investigate this relationship. As health care systems increasingly integrate telemedicine into routine care, understanding whether patient satisfaction differs between virtual and in-person follow-up, and how digital literacy shapes this experience, is essential for guiding clinical practice and health policy [14]. Accordingly, this randomized controlled trial aimed to compare patient satisfaction between telemedicine and in-person postoperative follow-up after upper-extremity surgery in an urban Thai population and to examine the association between digital literacy and patient satisfaction. We hypothesized that telemedicine would result in no significant difference in patient satisfaction compared with traditional in-person follow-up and that higher levels of digital literacy would be associated with greater patient satisfaction.

## Methods

### Ethical Considerations

This study was approved by the institutional review board of Vajira Hospital, Faculty of Medicine Vajira Hospital, Navamindradhiraj University (COA 012/2568) and conducted in accordance with the Declaration of Helsinki. Written informed consent was obtained from all participants prior to enrollment.

All data were deidentified before analysis and stored in secure, password-protected hospital systems accessible only to authorized researchers. Telemedicine visits were conducted using a secure, hospital-based platform.

Participants received a modest compensation of THB 100 (US $2.7) upon completion of the 6-week postoperative follow-up visit.

### Trial Registration

The study protocol was retrospectively registered with the Thai Clinical Trials Registry (TCTR20250528008) on May 28, 2025, after participant enrollment had begun but before completion of recruitment. Participant screening and enrollment occurred between February and August 2025, with the first participant enrolled on February 2, 2025. No changes were made to the study objectives, outcomes, or planned analyses after trial initiation or registration.

The study protocol, including the study objectives, eligibility criteria, outcome measures, and planned statistical analyses, had been finalized prior to the enrollment of the first participant and remained unchanged throughout the study. Although trial registration occurred after enrollment had begun due to administrative delays, no modifications were made to the study design, outcomes, or analyses after trial initiation or registration.

### Study Design

This study was a single-center, open-label, parallel-group randomized controlled trial with a 1:1 allocation ratio comparing telemedicine follow-up with conventional in-person outpatient department (OPD) follow-up after nontraumatic hand and upper-extremity surgery. The trial was conducted at the Department of Orthopedics, Faculty of Medicine Vajira Hospital, Navamindradhiraj University, Bangkok, Thailand. Randomization was performed by an independent coordinator using a computerized block randomization sequence with a fixed block size of 4 to ensure balanced group allocation.

### Participants

Adults aged 18 to 80 years who underwent elective, nontraumatic hand or upper-extremity surgery were eligible for inclusion. Diagnoses were categorized using *International Classification of Diseases, Tenth Revision* (*ICD-10*) codes related to upper-extremity conditions and procedures involving the shoulder, arm, elbow, forearm, wrist, and hand. Eligible participants resided in Bangkok or neighboring urban provinces (Nonthaburi, Pathum Thani, Nakhon Pathom, Samut Prakan, Samut Sakhon, and Chachoengsao) and had access to a smartphone, tablet, or computer with a stable internet connection to support telemedicine follow-up.

Exclusion criteria included (1) refusal to participate, (2) conditions requiring radiographic evaluation or direct physical examination at follow-up, as determined by the treating surgeon, and (3) cognitive, visual, or physical limitations that would prevent completion of telemedicine or questionnaire-based assessments. All diagnoses and eligibility determinations were confirmed by a board-certified orthopedic hand surgeon.

Participants were informed that they could withdraw from the study at any time, and those in the telemedicine group could be transitioned to in-person care if clinically indicated by their attending physician.

### Intervention and Follow-Up Protocol

Participants were randomized to receive either telemedicine or in-person outpatient (OPD) follow-up at 2 and 6 weeks postoperatively. All participants received standardized postoperative education during their 2-week clinic visit, including wound care instructions, rehabilitation guidance, and information on potential complications.

Participants allocated to the telemedicine group received an additional structured, in-person training session provided by the Department of Telemedicine at the Faculty of Medicine Vajira Hospital. This training covered installation and login procedures for Vajira@Home, the hospital’s dedicated telemedicine app, as well as demonstrations on how to navigate the interface, position the camera for optimal wound visualization, and complete a secure virtual follow-up visit (Multimedia Appendix 1). This brief training ensured that participants were capable of performing their telemedicine consultation independently from home using their own devices and internet connection.

At the 6-week follow-up, participants in the telemedicine group completed their visit through a secure hospital-based videoconferencing platform integrated within the Vajira@Home app. Participants in the OPD group returned for a conventional in-person clinic visit. Both groups followed identical clinical assessment protocols, including history taking, evaluation of wound healing, visual or in-person physical examination (with range-of-motion assessment when appropriate), and standardized documentation. Any findings requiring urgent or additional care were managed according to routine postoperative practice. Telemedicine participants could be converted to in-person evaluation at the discretion of the attending hand surgeon.

### Outcome Measures

The primary outcome was overall patient satisfaction at 6 weeks postoperatively, assessed using a validated 6-item Likert scale questionnaire (1=strongly disagree to 5=strongly agree). Satisfaction was also assessed at 2 weeks to evaluate changes over time. The questionnaire covered 4 domains: communication quality, convenience, understanding of postoperative care, and overall satisfaction. Domain-specific and total scores were analyzed, with higher scores indicating greater satisfaction.

The secondary outcome was digital literacy, measured at baseline using a standardized instrument adapted from the Digital Literacy Scale [15]. Participants rated three items assessing (1) fluency in using digital devices, (2) ability to use technology for telemedicine purposes, and (3) comfort communicating with physicians through digital tools. Each item was scored on a 5-point Likert scale, yielding a total score ranging from 3 to 15, with higher scores indicating greater digital literacy. Total scores were also converted to percentage values and categorized as poor (<60%), moderate (60%‐80%), or good (>80%) according to Bloom taxonomy thresholds.

The questionnaire was translated from English into Thai by certified professional translators and reviewed by English-language faculty members to ensure linguistic accuracy and conceptual equivalence. Internal consistency reliability of the adapted instrument was assessed using Cronbach alpha, demonstrating good reliability.

### Sample Size Calculation

Given the exploratory nature of this study and the absence of prior effect size estimates in comparable populations, a formal a priori sample size calculation based on a predefined effect size or noninferiority margin was not performed. Instead, a pragmatic sample size approach was adopted, informed by previously published telemedicine studies in orthopedic postoperative care that consistently reported high levels of patient satisfaction with minimal between-group differences.

Based on a prior randomized study by Cha et al [16] evaluating telemedicine follow-up after shoulder arthroscopy, overall patient satisfaction rates exceeding 90% were observed in both telemedicine and in-person groups. Assuming similarly high satisfaction proportions and small absolute differences between groups, a total sample size of approximately 64 participants (32 per group) was considered sufficient to allow exploratory between-group comparisons at a 2-sided significance level of .05.

To account for a potential dropout rate of approximately 5%, the target sample size was increased to 70 participants, with 35 participants allocated to each group.

Post hoc consideration suggests that, given the observed ceiling effects in satisfaction scores, the study may have been underpowered to detect small but clinically meaningful differences between follow-up modalities. Assuming a small effect size (Cohen *d* of approximately 0.3), a substantially larger sample size would have been required to achieve 80% statistical power. Therefore, the findings should be interpreted as exploratory, and larger studies are warranted to confirm these results.

### Data Collection and Risk Mitigation

Sociodemographic data collected at enrollment included age, sex, education, occupation, income, residence, and health care coverage. Technical readiness (device ownership, internet access, and privacy for consultations) was also recorded. To minimize risks related to privacy breaches, all data were stored in encrypted, password-protected hospital systems accessible only to authorized researchers. Participants received training in the use of the telemedicine app to prevent errors or delays during virtual sessions.

### Blinding

This study was a nonblinded randomized controlled trial, as blinding of participants and clinicians was not feasible due to the nature of the interventions. Participants were aware of their assigned follow-up modality (telemedicine or in-person OPD), and the treating orthopedic surgeons could not be blinded because they directly conducted both types of follow-up assessments.

To minimize potential bias, both groups received identical postoperative education at the 2-week visit, and follow-up assessments followed a standardized clinical protocol, including structured history taking, wound evaluation, range-of-motion assessment when appropriate, and uniform documentation. Patient satisfaction and digital literacy outcomes were collected using validated, self-administered questionnaires, reducing the risk of measurement bias.

### Statistical Analysis

Data were analyzed using SPSS Statistics (version 30; IBM Corp) [17]. Continuous variables are presented as mean (SD) or median (IQR), depending on data distribution. Normality was assessed using the Shapiro-Wilk test. Between-group comparisons of continuous variables were performed using the 2-tailed independent-samples *t* test or the Mann-Whitney *U* test, as appropriate. Categorical variables were analyzed using the chi-square test or Fisher exact test. Because questionnaire responses were measured using Likert scales, nonparametric tests were applied for these variables. Between-group comparisons were conducted using the Mann-Whitney *U* test, and within-group comparisons between week 2 and week 6 satisfaction scores were assessed using the Wilcoxon signed-rank test. A 2-sided *P* value <.05 was considered statistically significant. All analyses followed the intention-to-treat principle.

## Results

### Overview

**Figure 1.**
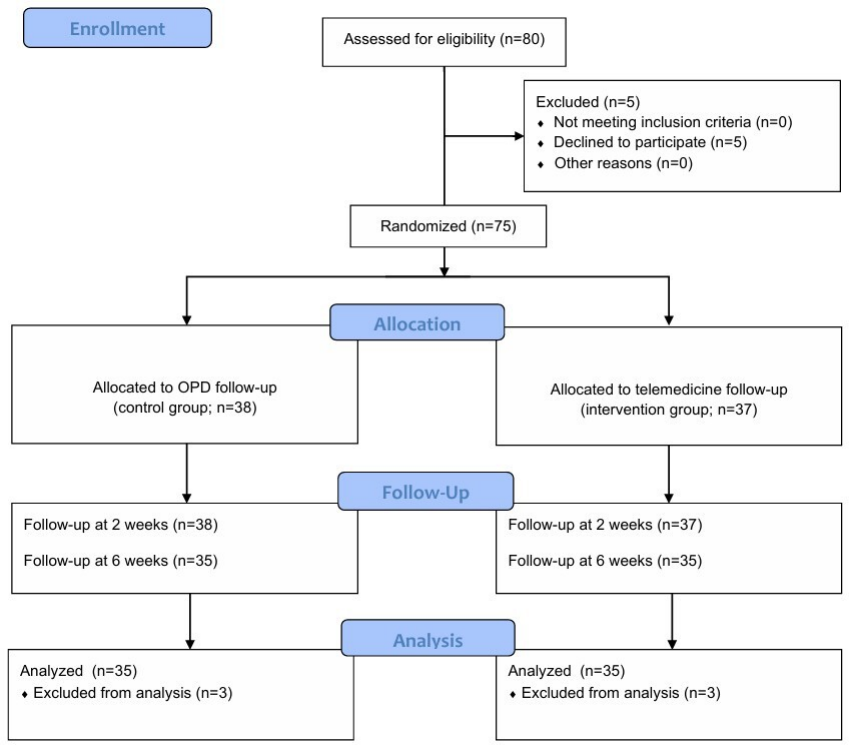
CONSORT (Consolidated Standards of Reporting Trials) flow diagram. OPD: outpatient department.

Participants were recruited between February and August 2025, during which all scheduled 2-week and 6-week follow-up assessments were completed. No external or secular events occurred during the study period that could have influenced recruitment, access to telemedicine services, or follow-up procedures. Of the 75 patients screened for eligibility, 93.3% (n=70) were enrolled and randomized equally to the telemedicine group (n=35, 50%) and the OPD group (n=35, 50%). Participant flow through the trial, including enrollment, allocation, follow-up, and analysis, is presented in Figure 1. No participants were lost during follow-up. All randomized participants completed their assigned follow-up modality at both time points, resulting in consistent denominators (n=70) for all primary analyses. Because all participants adhered to and used their assigned follow-up modality as intended (telemedicine: 35/35, 100% virtual visits; OPD: 35/35, 100% in-person visits), analyses were conducted according to the intention-to-treat principle, and no additional per-protocol or user-only analyses were required.

Baseline characteristics are presented in Table 1. Participants in the telemedicine group were younger on average than those in the OPD group (mean 53.3, SD 11.5 vs mean 59.7, SD 14.2 y) and had slightly higher digital literacy scores. Other demographic and clinical variables—including sex, diagnosis, geographic origin, income, employment, and health care coverage—were broadly similar between groups. Educational attainment differed modestly, with a greater proportion of participants in the OPD group holding undergraduate or postgraduate degrees. Consultation duration appeared longer in the telemedicine group (median 15, IQR 11.25-18.75 min) than in the OPD group (median 10, IQR 7.5-12.5 min).

**Table 1.** Baseline characteristics and technical access of study participants^a^.

Characteristic	Telemedicine group (n=35)	OPD^b^ group (n=35)
Age (y), mean (SD)	53.3 (11.5)	59.7 (14.2)
Male, n (%)	12 (34.3)	15 (42.9)
Female, n (%)	23 (65.7)	20 (57.1)
Carpal tunnel syndrome, n (%)	11 (31.4)	8 (22.9)
Trigger finger, n (%)	13 (37.1)	17 (48.6)
De Quervain tenosynovitis, n (%)	4 (11.4)	1 (2.9)
Other diagnoses, n (%)	7 (20)	9 (25.7)
Bangkok, n (%)	27 (77.1)	22 (63.9)
Metropolitan area outside Bangkok, n (%)	8 (22.9)	13 (37.1)
High school education or lower, n (%)	17 (48.6)	11 (31.4)
Undergraduate education or higher, n (%)	18 (51.4)	24 (68.6)
Universal Coverage Scheme, n (%)	8 (22.9)	9 (25.7)
Social Security Scheme, n (%)	13 (37.1)	4 (11.4)
Civil Service Medical Benefits Scheme, n (%)	10 (28.6)	16 (45.7)
Group or private insurance, n (%)	2 (5.7)	2 (5.7)
None, n (%)	2 (5.7)	4 (11.4)
Employed participants, n (%)	28 (80)	23 (65.7)
Unemployed participants, n (%)	7 (20)	12 (34.3)
Consultation duration (min), median (IQR)	15 (7.5)	10 (5.0)
Own a computer, n (%)	17 (48.6)	21 (60)
Own a smartphone, n (%)	35 (100)	35 (100)
Own a tablet, n (%)	13 (37.1)	15 (42.9)
Wi-Fi access at home, n (%)	32 (91.4)	32 (91.4)
Video chat device availability, n (%)	34 (97.1)	31 (88.6)
Able to log in successfully, n (%)	32 (91.4)	31 (88.6)
Private space for visit, n (%)	34 (97.1)	33 (94.3)

a Statistical tests were performed for between-group comparisons. Continuous variables were analyzed using the independent-samples *t* test or Mann-Whitney *U* test, as appropriate, and categorical variables were analyzed using the chi-square test or Fisher exact test. Corresponding *P* values are not presented in this table.

b OPD: outpatient department.

### Digital Literacy and Technological Readiness

All participants owned smartphones and had stable internet access. Ownership of computers and tablets was similar between groups. As shown in Table 1, most participants reported having home Wi-Fi access and a private space suitable for virtual consultations (telemedicine group: 34/35, 97.1%; OPD group: 33/35, 94.3%; *P*=.10). Successful login to the hospital’s telemedicine platform was achieved by 91.4% (32/35) of participants in the telemedicine group and 88.6% (31/35) in the OPD group (*P*=.56).

Although the categorical distribution of digital literacy levels did not differ significantly between groups (*P*=.10), the telemedicine group had a significantly higher mean digital literacy score than the OPD group (mean 12.0, SD 3.5 vs mean 10.2, SD 2.5; *P*=.02; Table 2).

Age-stratified analyses of individual digital literacy items showed no statistically significant differences between groups across all domains, including device fluency, telemedicine ability, and comfort communicating with physicians (all *P*>.05; Table 3).

**Table 2.** Digital literacy levels^a^.

Variable	Telemedicine group, n (%)^b^	Outpatient department group, n (%)^c^	*P* value^d^
Poor (score 3-8)	6 (17.1)	6 (17.1)	—^e^
Moderate (score 9-11)	8 (22.9)	16 (45.7)	—
Good (score 12-15)	21 (60.0)	13 (37.1)	—

a Digital literacy was assessed using a 3-item Likert scale (1=strongly disagree to 5=strongly agree; total score range 3‐15), with higher scores indicating greater digital literacy. Total scores were analyzed as continuous variables using independent-samples *t* tests. Individual Likert scale items were compared using the Mann-Whitney *U* test. Categorical variables were analyzed using the chi-square test or Fisher exact test, as appropriate.

b Total score: mean 12.0 (SD 3.5).

c Total score: mean 10.2 (SD 2.5).

d Total score: *P*=.02.

e Not applicable because at least one group contained no participants in this category, preventing statistical comparison.

**Table 3.** Age-stratified digital literacy scores^a^.

Item	Telemedicine group (<60 y), mean (SD)	OPD^b^ group (<60 y), mean (SD)	*P* value	Telemedicine group (≥60 y), mean (SD)	OPD group (≥60 y), mean (SD)	*P* value
Device fluency	4.00 (1.12)	3.94 (0.90)	.83	3.30 (1.70)	3.17 (1.16)	.91
Telemedicine ability	4.00 (1.15)	3.65 (0.86)	.85	3.50 (1.72)	3.06 (1.14)	.88
Comfort communicating	4.36 (1.19)	4.00 (0.79)	.76	4.20 (1.32)	3.47 (1.28)	.86

a Individual Likert scale items were compared using the Mann-Whitney *U* test. Categorical variables were analyzed using the chi-square test or Fisher exact test, as appropriate.

b OPD: outpatient department.

### Patient Satisfaction

Table 4 Patient satisfaction remained uniformly high at both postoperative follow-up assessments. At 2 weeks, the median overall satisfaction score was 5 (IQR 5-5) in both the telemedicine and OPD groups, with 94.3% (33/35) of participants in each group assigning the maximum score of 5 (P=.99). At 6 weeks, the median overall satisfaction score also remained 5 (IQR 5-5) in both groups. All participants in the telemedicine group (35/35, 100%) reported being fully informed about their postoperative care plans, compared with 94.3% (33/35) in the OPD group (P=.49). Ratings for surgeon communication, professionalism, and overall interaction quality were similarly high in both groups, with no statistically significant between-group differences observed (Table 4).

At 6 weeks, satisfaction levels remained unchanged. All telemedicine participants rated their overall care, visit experience, and surgeon interaction at the maximum score of 5. In the OPD group (n=35), 1 (2.9%) participant rated overall care as 4, while the remaining participants (n=34, 97.1%) gave a score of 5. Between-group comparisons revealed no statistically significant differences across any satisfaction domain (*P* values ranged from .15 to >.99).

**Table 4.** Patient satisfaction scores at 2 and 6 weeks postoperatively, stratified by follow-up modality and age group^a^.

Satisfaction item, time, and score			Telemedicine group (<60 y; n=25), n (%)	OPD^b^ group (<60 y; n=17), n (%)	*P* value (<60 y)	Telemedicine group (≥60 y; n=10), n (%)	OPD group (≥60 y; n=18), n (%)	*P* value (≥60 y)
Overall care satisfaction								
2 wk								
Score ≤4			1 (4)	1 (5.9)	>.99	1 (10)	1 (5.6)	>.99
Score 5			24 (96)	16 (94.1)	—^c^	9 (90)	17 (94.4)	—
6 wk								
Score ≤4			0 (0)	1 (5.9)	.41	0 (0)	0 (0)	>.99
Score 5			25 (100)	16 (94.1)	—	10 (100)	18 (100)	—
Satisfaction with follow-up visit								
2 wk								
Score ≤4			1 (4)	1 (5.9)	>.99	1 (10)	1 (5.6)	>.99
Score 5			24 (96)	16 (94.1)	—	9 (90)	17 (94.4)	—
6 wk								
Score ≤4			0 (0)	0 (0)	>.99	0 (0)	0 (0)	>.99
Score 5			25 (100)	17 (100)	—	10 (100)	18 (100)	—
Information about postoperative care								
2 wk								
Score ≤4			0 (0)	2 (11.8)	.16	0 (0)	0 (0)	>.99
Score 5			25 (100)	15 (88.2)	—	10 (100)	18 (100)	—
6 wk								
Score ≤4			0 (0)	1 (5.9)	.41	0 (0)	0 (0)	>.99
Score 5			25 (100)	16 (94.1)	—	10 (100)	18 (100)	—
Satisfaction with surgeon								
2 wk								
Score ≤4			2 (8)	2 (11.8)	>.99	0 (0)	0 (0)	>.99
Score 5			23 (92)	15 (88.2)	—	10 (100)	18 (100)	—
6 wk								
Score ≤4			1 (4)	1 (5.9)	>.99	0 (0)	0 (0)	>.99
Score 5			24 (96)	16 (94.1)	—	10 (100)	18 (100)	—
Recommendation to others								
2 wk								
Score ≤4			1 (4)	0 (0)	>.99	1 (10)	1 (5.6)	>.99
Score 5			24 (96)	17 (100)	—	9 (90)	17 (94.4)	—
6 wk								
Score ≤4			2 (8)	0 (0)	.52	0 (0)	0 (0)	>.99
Score 5			23 (92)	17 (100)	—	10 (100)	18 (100)	—
Overall surgeon rating								
2 wk								
Score ≤4			0 (0)	0 (0)	>.99	1 (10)	0 (0)	.36
Score 5			25 (100)	17 (100)	—	9 (90)	18 (100)	—
6 wk								
Score ≤4			0 (0)	0 (0)	>.99	0 (0)	0 (0)	>.99
Score 5			25 (100)	17 (100)	—	10 (100)	18 (100)	—

a Satisfaction was assessed using a validated 6-item Likert scale (1=strongly disagree to 5=strongly agree). No scores below 4 were reported. Between-group comparisons were performed using the chi-square test or Fisher exact test, as appropriate.

b OPD: outpatient department.

c Not applicable because all participants reported the same score, resulting in no variability for statistical comparison.

### Longitudinal Satisfaction Trends

Within-group analyses comparing week 2 and week 6 follow-up assessments showed consistent satisfaction over time in both cohorts (Table 4). In the telemedicine group, mean changes across individual satisfaction items ranged from 0.00 to 0.10, none of which were statistically significant (all *P*>.05). The OPD group exhibited similarly small variations (mean difference ≤0.06; *P*>.30). These findings indicate that satisfaction remained stable throughout the postoperative period, with no evidence of decline in either group.

### Summary of Findings

Overall, telemedicine follow-up produced patient satisfaction levels that were not significantly different from those of traditional in-person outpatient visits. Although participants in the telemedicine group were younger and demonstrated slightly higher digital literacy, these differences did not adversely affect satisfaction outcomes. Both models of care were highly acceptable to patients, underscoring telemedicine as a well-accepted and feasible option for postoperative follow-up in appropriately selected patients undergoing upper-extremity surgery.

## Discussion

### Principal Findings

This randomized controlled trial evaluated patient satisfaction with telemedicine versus conventional in-person OPD follow-up after upper-extremity surgery and examined whether digital literacy influenced satisfaction. Patient satisfaction was uniformly high in both groups at 2 and 6 weeks postoperatively, with no significant differences across satisfaction domains. Although participants in the telemedicine group were younger and had slightly higher digital literacy scores, no significant association was observed between digital literacy and satisfaction. These findings suggest that telemedicine follow-up, when supported by brief in-person app instruction, provides a patient experience that is not significantly different from traditional postoperative care. However, this study was not designed to evaluate clinical safety, complication rates, unplanned visits, or efficiency-related outcomes, such as time savings or cost reduction; therefore, conclusions regarding safety or efficiency should not be inferred from these results.

### Interpretation and Comparison with Existing Literature

The absence of statistically significant differences in satisfaction between telemedicine and in-person follow-up is consistent with prior orthopedic studies demonstrating similar levels of patient-reported satisfaction across follow-up modalities [5,17,18]. Previous research in shoulder, knee, hip, and hand surgery has shown that telemedicine can maintain high satisfaction while improving convenience and reducing travel burden [4,19-21]. Our findings extend this evidence to postoperative upper-extremity care in an urban, middle-income country context.

While most telemedicine studies originate from high-income settings [7,18], this study was conducted in Thailand, where variability in digital literacy and access to technology may present implementation challenges [11,13]. Despite this, satisfaction remained high in both groups, suggesting that telemedicine can be effectively integrated into postoperative care when supported by appropriate institutional infrastructure and patient education. A key contribution of this study is the evaluation of digital literacy as a potential determinant of satisfaction. Although digital literacy scores differed slightly between groups, no significant association with satisfaction was observed. This finding suggests that factors such as effective communication, structured postoperative education, and continuity of care may be more influential than baseline digital competence [10,11]. The brief in-person orientation to the telemedicine app may have mitigated usability barriers and supported equitable engagement with virtual follow-up.

### Limitations

This study has several limitations. Its single-center design in an urban setting may limit generalizability to rural or resource-limited populations. In addition, the study cohort consisted predominantly of urban residents with universal smartphone ownership and stable internet access, which may not reflect the circumstances of rural populations, older adults, or individuals with lower digital literacy or limited access to digital infrastructure. Implementation of telemedicine in such settings may require additional support strategies, including simplified user interfaces, extended in-person training, caregiver involvement, or hybrid care models combining virtual and in-person follow-up.

The modest sample size limited subgroup analyses according to age, educational level, or degree of digital literacy. In addition, patient satisfaction was assessed using Likert scale questionnaires, which demonstrated a pronounced ceiling effect, with most participants reporting maximum satisfaction scores across domains. Although this reflects a highly positive overall patient experience, such score clustering reduces the sensitivity of the instrument to detect subtle yet clinically meaningful differences between follow-up modalities. As a result, the absence of statistically significant differences should be interpreted as indicating similarly high overall satisfaction rather than complete equivalence across all experiential dimensions. Future studies may benefit from more nuanced or modality-specific satisfaction measures, as well as alternative metrics such as the Net Promoter Score, to better distinguish levels of patient experience among highly satisfied populations.

In addition, trial registration was completed after participant enrollment had begun. Although the study protocol, including objectives, eligibility criteria, outcome measures, and planned analyses, was finalized prior to enrollment and remained unchanged throughout the study, the timing of registration should be considered a limitation and may raise concerns regarding transparency. Finally, this study focused primarily on satisfaction outcomes and did not evaluate clinical outcomes, complication rates, unplanned visits, or efficiency-related metrics, such as time or cost savings, which should be addressed in future research.

### Implications for Future Research

Future multicenter studies involving diverse populations are needed to enhance generalizability. Qualitative methods may provide deeper insights into patient experiences with telemedicine, particularly regarding communication, trust, and usability [10]. Incorporation of clinical outcomes, economic evaluations, and longer-term follow-up would further clarify the role of telemedicine in postoperative orthopedic care.

### Conclusions

Telemedicine represents a well-accepted and feasible alternative to traditional in-person follow-up after upper-extremity surgery. The consistently high satisfaction observed across both follow-up modalities suggests that, when supported by adequate digital infrastructure and patient education, telemedicine can be effectively integrated into postoperative care pathways. The observed benefits suggest potential systemic efficiency—such as reduced outpatient clinic burden—and improved patient convenience, including decreased travel and waiting time, as supported by prior research; however, clinical safety outcomes, economic efficiency, and cost-effectiveness were not directly evaluated in this study.

## Supplementary material

10.2196/86918 Multimedia Appendix 1 Screenshots of the Vajira@Home telemedicine platform used for patient login, video consultation, and postoperative follow-up.

10.2196/86918 Checklist 1 CONSORT EHEALTH checklist (V1.6.1).

## Abbreviations

*ICD-10*: *International Classification of Diseases, Tenth Revision*
OPD: outpatient department
