# Supplementary figures and images for: Digital Literacy and Patient Satisfaction in Telemedicine Follow-Up With In-Person App Instruction Versus Outpatient Department Follow-Up After Upper-Extremity Surgery: A Randomized Controlled Trial
Source: J Med Internet Res. 2026 Jun 25;28:e86918. doi: 10.2196/86918 (PMC13299266; doi:10.2196/86918)

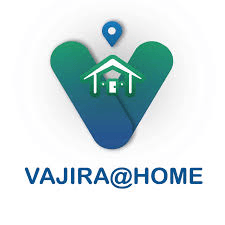

Supplement: Multimedia Appendix 1 [file jmir-v28-e86918-s001.png]
